# Molecularly Imprinted Filtering Adsorbents for Odor Sensing

**DOI:** 10.3390/s16111974

**Published:** 2016-11-23

**Authors:** Sho Shinohara, You Chiyomaru, Fumihiro Sassa, Chuanjun Liu, Kenshi Hayashi

**Affiliations:** 1 Graduate School of Systems Life Sciences, Kyushu University, 744 Motooka, Nishi-ku, Fukuoka 819-0395, Japan; s.shinohara.877@s.kyushu-u.ac.jp; 2 Graduate School of Information Science and Electrical Engineering, 744 Motooka, Nishi-ku, Fukuoka 819-0395, Japan; youchiyomaru@hotmail.co.jp (Y.C.); sassa@ed.kyushu-u.ac.jp (F.S.); liu_d@use-ebisu.co.jp (C.L.); 3 Research Laboratory, U.S.E Co. Ltd., 1-10-4 Hiroo, Shibuya-ku, Tokyo 150-0012, Japan

**Keywords:** odor sensor, adsorbents, molecularly imprinted filtering adsorbent

## Abstract

Versatile odor sensors that can discriminate among huge numbers of environmental odorants are desired in many fields, including robotics, environmental monitoring, and food production. However, odor sensors comparable to an animal’s nose have not yet been developed. An animal’s olfactory system recognizes odor clusters with specific molecular properties and uses this combinatorial information in odor discrimination. This suggests that measurement and clustering of odor molecular properties (e.g., polarity, size) using an artificial sensor is a promising approach to odor sensing. Here, adsorbents composed of composite materials with molecular recognition properties were developed for odor sensing. The selectivity of the sensor depends on the adsorbent materials, so specific polymeric materials with particular solubility parameters were chosen to adsorb odorants with various properties. The adsorption properties of the adsorbents could be modified by mixing adsorbent materials. Moreover, a novel molecularly imprinted filtering adsorbent (MIFA), composed of an adsorbent substrate covered with a molecularly imprinted polymer (MIP) layer, was developed to improve the odor molecular recognition ability. The combination of the adsorbent and MIP layer provided a higher specificity toward target molecules. The MIFA thus provides a useful technique for the design and control of adsorbents with adsorption properties specific to particular odor molecules.

## 1. Introduction

With progress in organic materials, electronics, and micro-fabrication technologies, a number of highly advanced odor sensors have been proposed. However, compared with physical or chemical sensors such as inertial [1] or blood glucose sensors [2], commercial application of these odor sensors is not widespread. A major reason for this is the complexity of the analytes that make up an odor. An odor usually consists of a mixture of several gases, and the possible number of combinations of these gases is enormous. One of the simplest approaches for the detection and discrimination of odors is to use a large number of odor-specific sensors in an array; this is known as the electronic nose (e-nose) approach. However, it is often impossible to prepare such large numbers of odor-specific sensors as an array. In contrast, it has recently been reported that the human nose can discriminate a single odor from over one trillion odors [3]. When animals smell odors, the olfactory bulb is activated by stimulation of the olfactory receptors. This information from the activated olfactory bulb is called an odor map [4,5], and it is composed of several hundreds of glomeruli on the olfactory bulb. Glomeruli activated by odorants with similar molecular characteristics are located close together, and form an odor cluster [6,7,8,9]. With the resulting cluster map, a huge number of odors can easily be discriminated.

The generation of odor cluster maps thus enables the measurement of odors in the same manner as that used in animal olfaction. Each cluster corresponds to a particular type of molecular structure information, for example functional groups, molecular sizes, or shapes, and recognizing the molecular structure of the odorant enables qualitative and quantitative analysis of the odor based on its visible pattern information [10,11,12]. Thus, the design and flexible control of the sensor specificity toward parameters based on odor cluster maps are important factors for odor sensor development.

**Figure 1:**
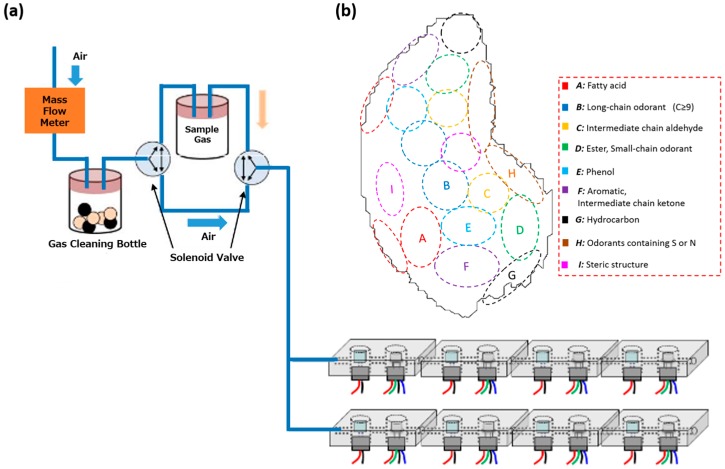
Conceptual approach for a bio-inspired odor sensing system. (**a**) Adsorption-separation odor sensing system; (**b**) resulting odor cluster map for odor evaluation [13].

In our previous study, we developed a gas sensing system aimed at recognizing complex molecular structures using a gas concentrating-separating sensing device [13]. It was possible to regenerate odor maps from sensor responses to odorants using this system. The system had eight sensing cells, and each cell consisted of an adsorbent separation unit and a gas sensing unit; the adsorbent separation unit contained an adsorbent film on a micro-ceramic heater, and the separated odorants were quantified with a gas sensing unit made up of a metal oxide semiconductor gas sensor. Figure 1 shows the conceptual design of this odor sensing system. The selectivity of this device depends on the molecular recognition properties of the adsorbents; it requires adsorbents with high selectivity in molecular recognition. The sensor system can selectively detect odors if it incorporates adsorbents with appropriate selectivities for various odorants. However, the ability of the previously used adsorbents was insufficient for the recognition of a wide variety of odor molecules.

In this study, a new molecular selective adsorbent for gas preconcentrator was developed by combinations of adsorbent materials and a molecularly imprinted polymer (MIP). First, we focused on polydimethylsiloxane (PDMS), which has superior fabrication processability and a high adsorption capacity and specificity for odorants, as confirmed in our previous work. In addition, other adsorbent polymers, such as divinylbenzene copolymer (DVB) and polyvinyl alcohol (PVA), were compounded into a matrix with a PDMS substrate, with the aim of controlling the solubility parameters of the adsorbents. A polyvinyl chloride (PVC)—containing plasticizer was also examined.

**Figure 2:**
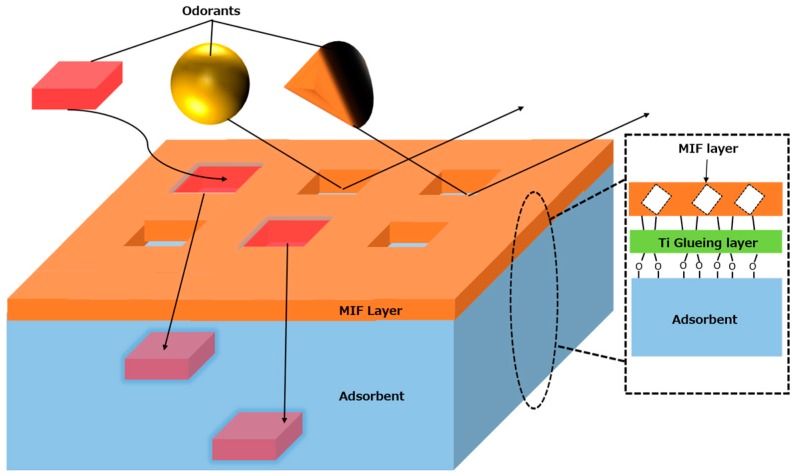
Structure of a MIFA.

To improve the selectivity of the adsorbents toward the odor molecules, the surface of the adsorbent substrates was modified with a molecularly imprinted filter (MIF), which is an MIP layer formed by a sol-gel method, in which the MIP retains molecular void space in its structure. The modified adsorbent is called a molecularly imprinted filtering adsorbent (MIFA). Figure 2 shows a structural schematic drawing of a MIFA. Only the molecules passing through this thin MIF film will be concentrated into the adsorbent substrate layer; this gives the composite molecular sieving properties. Consequently, gas-selective adsorbents can be developed using composite materials prepared from adsorbent polymers and MIP layers, and the adsorption properties can be designed and controlled using combinations of adsorbents and MIF layers specific to the target analytes. MIFAs are expected to have good adaptability for an odor-clustering sensing system.

## 2. Materials and Methods 

### 2.1. Preparation of Composite Adsorbents

PDMS-based composite adsorbents containing DVB (PDMS-DVB) and PVA (PDMS-PVA) were prepared. PDMS is known to be a good adsorbent for a broad range of odorant molecules, and can in particular adsorb large amounts of ketones. However, fatty acids and alcohols are not well adsorbed by PDMS because of its low hydrophilicity [13]. We therefore made a PDMS composite adsorbent containing DVB (2%), which was expected to adsorb fatty acids, and a composite with PVA (30%), which can adsorb alcohols, to modify the adsorption properties of PDMS.

We also prepared PVC-dioctyl phthalate (PVC-DOP) and polyethylene glycol-PVA (PEG-PVA) (1:1) composite adsorbents to enhance the adsorption of alcohols. PVC is a relatively hydrophilic material and its chemical and physical properties can be controlled by mixing this polymer with the hydrophobic plasticizing agent DOP. To control the gas adsorption properties by varying the hydrophobicity, DOP was mixed with PVC in ratios of 1:1, 1:2, 1:5, and 1:10. A mass of 7.5 g of each composite was dissolved in 5 mL tetrahydrofuran (THF) and stirred for 2 h. Films of the adsorbent substrates were then formed by evaporation of THF at room temperature. Finally, cured adsorbents were obtained.

The adsorbent materials used were chosen based on their solubility parameters, which are as follows: PDMS, 14.9; DVB, 9.3; PVA, 12.6; PVC, 9.5; and DOP, 12.1 (cal/cm^3^)^1/2^ [14]. 

### 2.2. Deposition of MIFs on the Adsorbent Substrates

To obtain sophisticated control over the adsorption properties of the adsorbents, a permselective filter was prepared by deposition of MIPs on the adsorbents. In this research, PVC-DOP, PDMS, PDMS-DVB, and PDMS-PVA were used as adsorbent substrates for the MIFAs. We developed two types of MIFs composed of from polyacrylic acid (PAA) and methacrylic acid (MAA).

**Figure 3:**
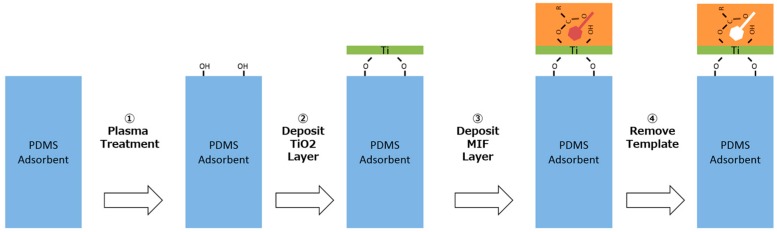
Procedure for MIFA fabrication.

The deposition method for the MIFA prepared using PAA (MIF_PAA_) is shown in Figure 3.

1. The polymer solution was prepared by mixing PAA (10 mM), HCl (50 mM), a template molecule (5 mM), and ethanol to obtain a certain concentration, and then stirring the mixture for 4 h.
2. The titanium alkoxide solution was prepared by mixing ethanol (1.5 mL), toluene (1.5 mL), and titanium(VI) butoxide (100 mM).
3. A gel layer of titanium oxide cannot be attached directly onto the PDMS surface because of its highly water-repellent nature. The surface of PDMS was therefore activated by hydrophilization by treatment with an O_2_ plasma (Harrick plasma, PDC-002).
4. A single titanium alkoxide layer was formed on the PDMS surface by dipping hydrophilization-treated PDMS into a titanium alkoxide solution for 20 min. Thereafter, the surface was rinsed with ethanol and dipped into the polymer solution to give a MIF_PAA_ film including the template molecule.
5. The template molecule was removed from the MIF by rinsing with ethanol and heating at 80 °C for 1 h.

In this way, an MIF_PAA_-coated adsorbent was obtained.

The MIF prepared using MAA (MIF_MAA_) was synthesized by copolymerization of polymerized MAA and a crosslinker in the presence of a template molecule. Here, ethylene glycol dimethacrylate (EGDMA) was used as the crosslinker. The deposition method for MIF_MAA_ is described below.

1. The prepolymer solution was prepared by mixing MAA (2 mmol), EGDMA (1 mmol), a template molecule (2 mmol), acetonitrile (40 mmol) as the diluent, and azobis-isobutyronitrile (2 mg) as the initiator and then stirring this mixture for 4 h.
2. A gel layer of titanium oxide was formed on the hydrophilization-treated PDMS using the same method as for the preparation of MIF_PAA_ [15]. The PDMS was then rinsed with ethanol and dipped into the prepolymer solution for 1 h at 80 °C.
3. The template molecule was removed as described above for MIF_PAA_.

In this way, an MIF_MAA_-coated adsorbent was obtained.

### 2.3. Characterization of the Adsorption Properties of the Adsorbent

To evaluate the specific adsorption of the adsorbent, we used gas chromatography–mass spectrometry (GC-MS) with a solid-phase microextraction (SPME) fiber auto-sampler [16], as described below.

**Figure 4:**
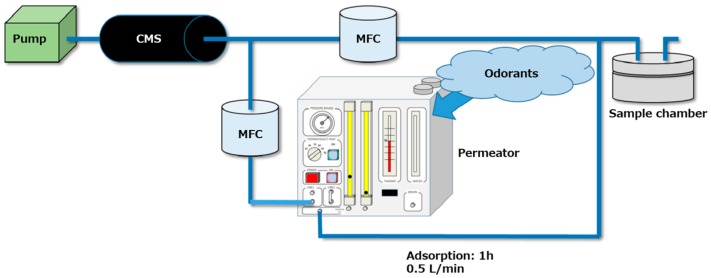
Adsorption experiment system. CMS: carbon molecular sieve column for obtaining clean air. MFC: mass flow controller. Sample adsorbents were placed in the sample chamber.

1. The adsorbents were placed in a sealed chamber and exposed to odorant gases (Figure 4). The odorants were volatilized at 50 °C in a permeator (PD-1B, GASTEC, Ayase, Japan) or a glass desiccator, and then left in the chamber for 1 h under gas flow at 0.5 L/min.
2. The adsorbents were transferred to screw tube vials and were introduced into the SPME auto-sampler (AOC5000 plus, Shimadzu, Japan) with an SPME fiber (23-gauge, 50/30 µm, DVB/CAR/PDMS, SPELCO, Bellefonte, PA, USA). The samples were then heated from 40 to 240 °C to desorb the odor molecules from the adsorbents. Finally, the amounts of the detached gases were determined using GC-MS (GCMS-QP2010, Shimadzu, Kyoto, Japan). This method is referred to below as the “GC-MS/SPME method”.

## 3. Results and Discussion

### 3.1. Influence of DOP Concentration on the Adsorption Properties of PVC-DOP Composite Adsorbents

**Figure 5:**
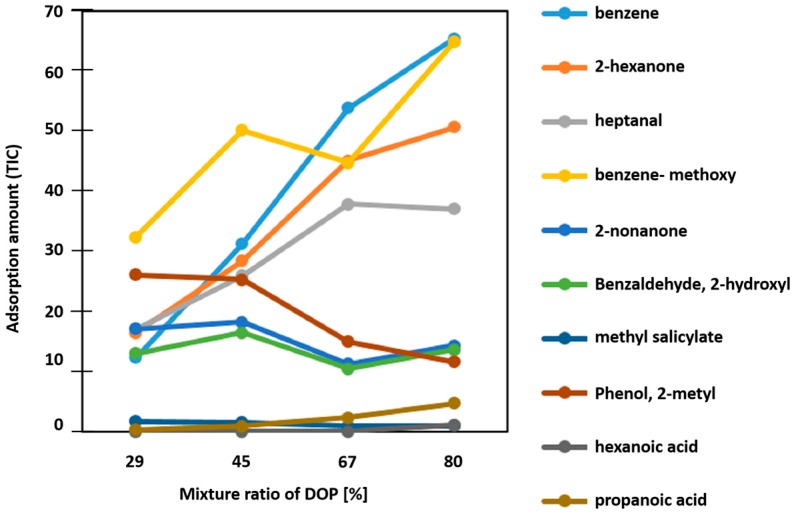
Adsorbed odorant amount (TIC: total ion current obtained from gas chromatography–mass spectrometry/solid-phase microextraction) of polyvinyl chloride–dioctyl phthalate (PVC-DOP) adsorbents as a function of DOP content.

**Figure 6:**
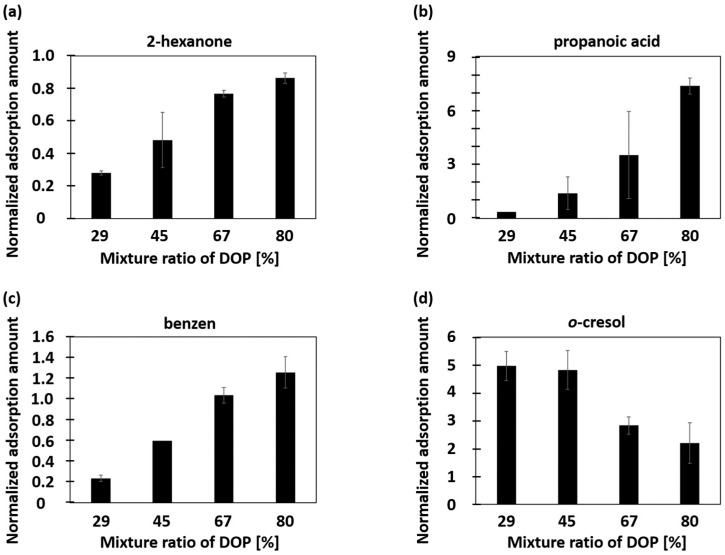
Adsorption properties of polyvinyl chloride–dioctyl phthalate (PVC-DOP) adsorbents as a function of DOP content, relative to those of polydimethylsiloxane (PDMS) adsorbents. Adsorption of (**a**) 2-hexanone; (**b**) propanoic acid; (**c**) benzene; (**d**) *o*-cresol.

The specific adsorption properties of PVC-DOP composite adsorbents with different mixing ratios were evaluated using the GC-MS/SPME method. In this experiment, 10 odorant gases (benzene, 2-hexanone, heptanal, anisole, 2-nonanone, propanoic acid, salicylaldehyde, methyl salicylate, *o*-cresol, hexanoic acid) were used as odor samples. These gases are typical bio-volatile organic compounds (BVOCs) from various chemical groups which are important analytes for food production and living environmental monitoring. Figure 5 illustrates the dependence of the amount of adsorbed gases on the DOP mixing ratio, and the amounts of adsorbed gas normalized to those adsorbed by a pure PDMS adsorbent are shown in Figure 6. The normalized values indicate whether the composite adsorbs greater amounts of gas than PDMS does. Figure 6 shows the individual values for 2-hexanone, propanoic acid, benzene, and *o*-cresol. The adsorbed gas amounts increased with the increasing DOP ratio for benzene, 2-hexanone, and propanoic acid; the change in the adsorbed amounts of propanoic acid is especially large. In contrast, the adsorbed amount of *o*-cresol decreased with the increasing DOP content. These specific absorption properties can be attributed to the change in solubility parameters on mixing DOP and PVC; hydrophobic adsorbents have a higher concentrating ability toward hydrophobic odorants. This result shows that the specific adsorption of gases by PVC-DOP composite adsorbents can be controlled by adjusting the mixing ratio. This composite is especially promising as an adsorbent for fatty acids.

### 3.2. Modification of the Adsorption Properties of PDMS by Addition of Other Adsorbents

**Figure 7:**
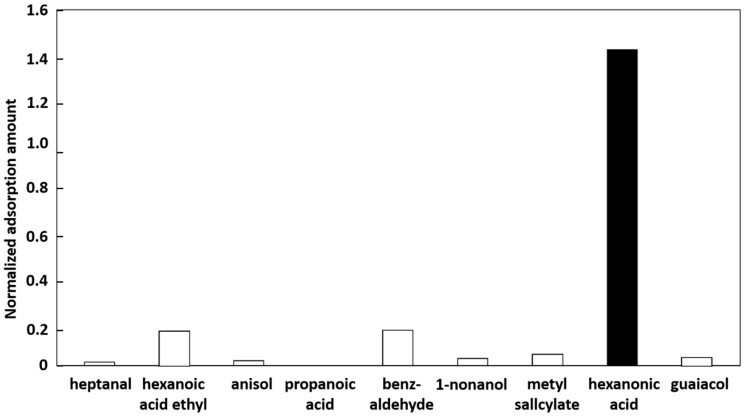
Amounts of gas adsorbed by a molecularly imprinted filtering adsorbent fabricated using hexanoic acid as the template. The amounts are normalized relative to the amounts of gas adsorbed by a polyvinyl chloride–dioctyl phthalate (PVC-DOP) adsorbent without a molecularly imprinted filter.

Next, the specific adsorption of PDMS mixed with other adsorbents (PVA and DVB) was evaluated using the GC-MS/SPME method. The PEG-PVA adsorbent was also evaluated. In this experiment, three odorant gases (2-heptanone, 1-heptanol and heptanoic acid) were used as the gas samples, and their structures are shown in Figure 7. The molecular structures of these three gases are similar and the lengths of their carbon chains are identical. Table 1 shows the normalized amounts of adsorbed gases relative to the amounts adsorbed by the pure PDMS adsorbent. Table 2 shows the adsorption ratios of 1-heptanol and heptanoic acid to 2-heptanone. The results indicate that PDMS-DVB and PEG-PVA are effective adsorbents for heptanoic acid, which is a fatty acid, and that PEG-PVA can be used for the alcohol 1-heptanol. 

The results described above show that the amount of gas adsorbed by PVC and PDMS adsorbents can be varied by mixing these adsorbents with materials that have different solubility parameters. In this way, composite adsorbents can be used to achieve rough selectivity toward different gases.

### 3.3. Controlling the Adsorption Properties of the PVC-DOP Adsorbent with a MIF

To control the specificity of the gas adsorption, a MIFA composed of a PVC-DOP adsorbent with a MIF_PAA_ layer was fabricated and its adsorption properties were evaluated. For this experiment, a PVC-DOP composite adsorbent containing 67% DOP was used. Two types of MIFAs were fabricated using different template materials (2-decanone and 2-nonanone), and an adsorbent without a MIF layer was used as a negative control. The amount of gas adsorbed by each adsorbent was measured using the GC-MS/SPME method. The sample gas consisted of a mixture of 2-decanone, 2-nonanone, hexanoic acid, nonanol, and 5-nonanone. For both MIFAs, the adsorption of the gas used as the template was increased compared with the amount adsorbed by the negative control (data not shown). In contrast, the amount of adsorbed non-template gases decreased, which shows that the MIF_PAA_ layers behave as MIFs. These results show that the specificity of the gas adsorption was enhanced by addition of MIFs to the adsorbents.

We also tested the gas specificity of the MIFA in detail using nine different gases (heptanal, hexanoic acid ethyl, anisole, propanoic acid, benzaldehyde, nonanol, methyl salicylate, hexanoic acid, and guaiacol). The MIFA fabricated with a hexanoic acid template was used in this experiment. Figure 7 shows the normalized amounts of adsorbed gas relative to those for the adsorbent without a MIF. The amount of gas adsorbed increased only for hexanoic acid, and decreased for all of the other gases. The normalized amount of adsorbed target gas, hexanoic acid, was seven times greater than the adsorbed amounts of the other gases.

**Figure 8:**
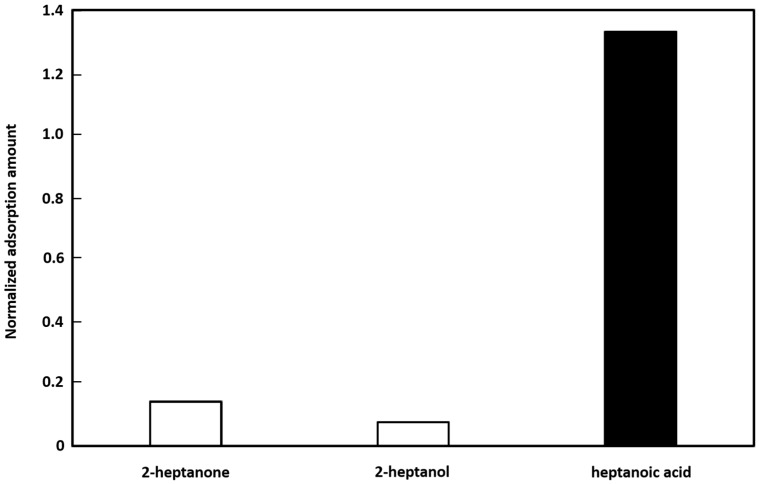
Adsorption specificity of a molecularly imprinted filtering adsorbent on a polydimethylsiloxane–divinylbenzene (PDMS-DVB) adsorbent layer. The template odorant was heptanoic acid.

The adsorption properties of PDMS-DVB adsorbents with a MIF_MAA_ filtering layer were also evaluated. The sample gases, 2-heptanone, 1-heptanol, and heptanoic acid, were adsorbed onto the MIFA, which was prepared using heptanoic acid as the template molecule. The adsorption properties were measured using the same method as used for the other experiments in this paper. A non-imprinted filtering adsorbent (NIFA), which is an adsorbent made following the same procedure as that used to prepare MIFAs but without a template molecule, was prepared as a negative control. The normalized amounts of adsorbed gas, relative to the amounts adsorbed with the NIFA, are shown in Figure 8. The normalized amount of adsorbed gas increased for heptanoic acid but decreased for 2-heptanone and 2-heptanol. The ratios between heptanoic acid and 2-heptanone, and heptanoic acid and 2-heptanol, were 9.5 and 18, respectively. These results show that the MIF layer could filter out a wide range of gas species. Thus, considerable control over the gas adsorption properties of adsorbents could be achieved. In our previous study, we confirmed the regeneration and degradation of MIF layers [17]. The sensing system using MIFAs has a heating unit, and then the adsorbed gas can be desorbed in a higher temperature condition (80 °C). Such desorption was confirmed before GC-MS/SPME evaluation, i.e., template gas molecules cannot be detected after the 80 °C vacuum-heating process. The lifetime of the MIF_PAA_ is 7 h under a high concentration gas of PAA solvent (ethanol). However, the MIFA can be used for several weeks in a normal gaseous concentration environment. In addition, the MIF layer can be refreshed and rebuilt in a reconfigurable process [17]. 

### 3.4. Comparison of the Filtration Efficiency of MIF_MAA_ and MIF_PAA_

**Figure 9:**
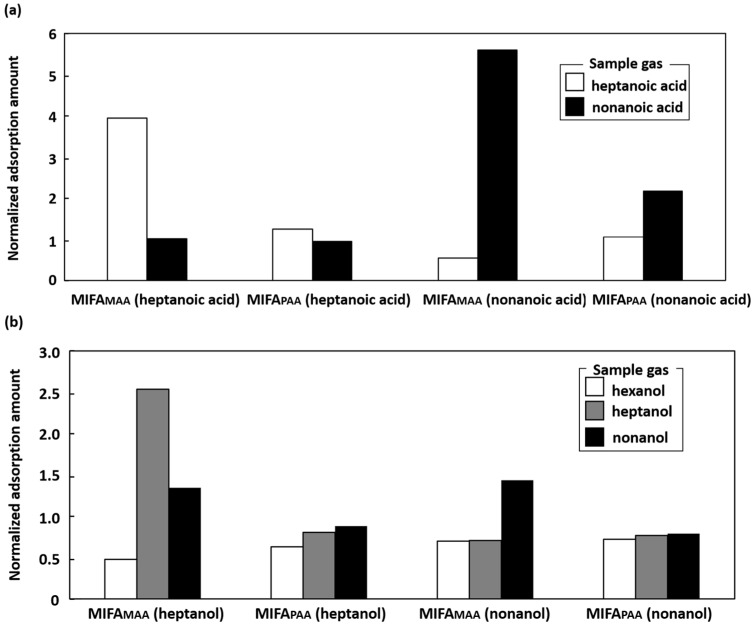
(**a**) Adsorption specificity of a molecularly imprinted filtering adsorbent (MIFA) for fatty acids; (**b**) Adsorption specificity of a MIFA for alcohols. Adsorbent samples are named as in the following example: MIFA_MAA_ (heptanol) = MIFA with a methacrylic acid (MAA)-MIF layer prepared using heptanol as the template.

To examine the filtration efficiencies of different MIF materials, the amounts of gases adsorbed by MIF_MAA_ and MIF_PAA_ were compared. In this experiment, both types of MIFs were deposited on a pure PDMS substrate. Two fatty acids (heptanoic acid and nonanoic acid) and two alcohols (heptanol and nonanol) were used as templates for the MIF layer. The normalized amounts of adsorbed gases, relative to the amount adsorbed by the NIFA, are shown in Figure 9. For both fatty acids and alcohols, MIF_MAA_ showed a better filtering performance than MIF_PAA_ did. MIF_MAA_ is believed to have specific adsorption sites in its fixed crosslinked structure, which make it a stable MIF. In contrast, MIF_PAA_ has flexibility and the fabrication of its loose MIF structure is easy [13]. Thus, the selection of MIF materials is another important factor in the design of a MIFA composite with high selectivity for specific odorants.

## 4. Conclusions

Highly selective gas adsorbents based on a MIFA were developed. The combination of a filter layer that is selective to specific molecules and a gas adsorbent with a high adsorption capacity and a rough gas selectivity enabled highly tunable control of the adsorption properties. The rough gas selectivity of the base adsorbent materials, PDMS and PVC, could be modified by mixing these materials with other polymer adsorbents, and these composite adsorbents functioned as broad gas-filtering materials. The combination of these composite adsorbents with MIFs gave greater control over their specific adsorption toward various odor molecules. Because of this high control over the adsorption properties, MIFAs are promising materials for the production of a set of odor sensors for the detection of clustered odorants. The set of sensors is a promising information source of a pattern recognition machine which usually requires a great deal of independent information for discrimination. The cluster maps were synthesized using the preliminary system [18]. This novel concept involving the use of a MIF provides a simple means by which to add molecular selectivity to other chemical sensors.

## Figures and Tables

**Table 1:** Adsorption specificity of composite adsorbents. Adsorption amounts (total ion current (TIC) values) were normalized to the TIC values of polydimethylsiloxane (PDMS).

	2-Heptanone	1-Heptanol	Heptanoic Acid
PDMS-DVB/pure PDMS	0.82	0.92	8.71
PEG-PVA/pure PDMS	0.3	1.1	6.33
PDMS-PVA/pure PDMS	1.0	1.08	3.02

**Table 2:** Adsorption specificity of composite adsorbents. Adsorption amounts determined from the total ion current (TIC values) were normalized to the TIC values for adsorbed 2-heptanone on each adsorbent.

	1-Heptanol/2-Heptanone	Heptanoic Acid/2-Heptanone
PDMS	0.479	0.00215
PDMS-DVB	0.541	0.0229
PEG-PVA	1.779	0.046
PDMS-PVA	0.515	0.00647

## References

[1] Updike S.J., Hicks G.P. (1967). The enzyme electrode. Nature.

[2] Yazdi N., Ayazi F., Najafi K. (1998). Micromachined inertial sensors. Proc. IEEE.

[3] Bushdid C., Magnasco M.O., Vosshall L.B., Keller A. (2014). Humans Can Discriminate More than 1 Trillion Olfactory Stimuli. Science.

[4] Mori K., Takahashi Y.K., Igarashi K.M., Yamaguchi M. (2006). Maps of odorant molecular features in the mammalian olfactory bulb. Physiol. Rev..

[5] Soucy E.R., Albeanu D.F., Fantana A.L., Murthy V.N., Meister M. (2009). Precision and diversity in an odor map on the olfactory bulb. Nat. Neurosci..

[6] Buck L.B. (2004). Olfactory receptors and odor coding in mammals. Nutr. Rev..

[7] Johnson B.A., Leon M. (2007). Chemotopic odorant coding in a mammalian olfactory system. J. Comp. Neurol..

[8] Matsumoto H., Kobayakawa K., Kobayakawa R., Tashiro T., Mori K., Sakano H. (2010). Spatial Arrangement of Glomerular Molecular-Feature Clusters in the Odorant-Receptor Class Domains of the Mouse Olfactory Bulb. J. Neurophysiol..

[9] Mori K., Yoshihara Y. (1995). Molecular recognition and olfactory processing in the mammalian olfactory system. Prog. Neurobiol..

[10] Gottfried J.A. (2009). Function follows form: Ecological constraints on odor codes and olfactory percepts. Curr. Opin. Neurobiol..

[11] Imahashi M., Hayashi K. (2012). Odor clustering and discrimination using an odor separating system. Sens. Actuators B Chem..

[12] Bozza T., Vassalli A., Fuss S., Zhang J.J., Weiland B., Pacifico R., Feinstein P., Mombaerts P. (2009). Mapping of Class I and Class II Odorant Receptors to Glomerular Domains by Two Distinct Types of Olfactory Sensory Neurons in the Mouse. Neuron.

[13] Imahashi M., Watanabe M., Jha S.K., Hayashi K. (2014). Olfaction-Inspired Sensing Using a Sensor System with Molecular Recognition and Optimal Classification Ability for Comprehensive Detection of Gases. Sensors.

[14] Plastic Material Dictionary. http://www.plastics-material.com/.

[15] Kunitake T., Lee S.W. (2004). Molecular imprinting in ultrathin titania gel films via surface sol-gel process. Anal. Chim. Acta.

[16] Snow N.H., Slack G.C. (2002). Head-space analysis in modern gas chromatography. TrAC Trends Anal. Chem..

[17] Imahashi M., Hayashi K. (2013). Concentrating materials covered by molecular imprinted nanofiltration layer with reconfigurability prepared by a surface sol–gel process for gas-selective detection. J. Colloid Interface Sci..

[18] Imahashi M., Kenshi H. (2014). Odor Clustering Based on Molecular Parameter for Odor Sensing. Sens. Mater..

